# A270 RISK FACTORS FOR COLORECTAL NEOPLASIA DEVELOPMENT IN INFLAMMATORY BOWEL DISEASE PATIENTS WITH PRIMARY SCLEROSING CHOLANGITIS: A CASE-CONTROL STUDY

**DOI:** 10.1093/jcag/gwad061.270

**Published:** 2024-02-14

**Authors:** L van Lierop, M Derks, M te Groen, C Tran, E Lytvyak, A Montano Loza, F Hoentjen

**Affiliations:** Medicine, University of Alberta, Edmonton, AB, Canada; Radboudumc, Nijmegen, Gelderland, Netherlands; Radboudumc, Nijmegen, Gelderland, Netherlands; Medicine, University of Alberta, Edmonton, AB, Canada; University of Alberta Faculty of Medicine & Dentistry, Edmonton, AB, Canada; Medicine, University of Alberta, Edmonton, AB, Canada; Medicine, University of Alberta, Edmonton, AB, Canada

## Abstract

**Background:**

Patients with inflammatory bowel disease (IBD) and concomitant primary sclerosing cholangitis (PSC-IBD) have a 25-year cumulative risk of up to 50% to develop colorectal neoplasia (CRN). Mucosal inflammation is an important driver of CRN development in IBD, but it is unknown whether this also applies to PSC-IBD patients.

**Aims:**

We aimed to assess the impact of active mucosal inflammation per colonic segment and endoscopic remission on CRN risk.

**Methods:**

This is a single-center case-control study, including cases with PSC-IBD with CRN, and controls with PSC-IBD without CRN. Exclusion criteria were a CRN diagnosis prior to IBD or PSC diagnosis, familial CRC syndromes, and insufficient endoscopic follow-up (ampersand:003C2 available colonoscopy reports, and for cases, ampersand:003C2 available endoscopy reports before CRN diagnosis). Data was collected on demographics, IBD and PSC disease characteristics, and IBD medication use. Data from all available colonoscopy and sigmoidoscopy reports between 2002 and 2023, including endoscopic and histologic disease severity, disease extent, and CRN lesions, was collected. The primary outcome was a diagnosis of CRN, secondary outcomes included CRN grade and location, inflammatory burden per colonic segment harboring CRN, and colonic surgery.

**Results:**

We included 247 PSC-IBD patients (163 ulcerative colitis, 80 Crohn’s disease) with a median follow-up of 6.3 years (IQR: 4.1-12.6) and 6 endoscopic procedures (IQR: 4.0-8.0). Median disease duration was 19 years for IBD (IQR: 11.0-26.0) and 13 years for PSC (IQR: 7.0-18.0). In 50 cases a total of 113 CRN lesions were observed, of which 11 (10%) were advanced CRN (aCRN; HGD or CRC). 23 patients (45%) were diagnosed with ampersand:003E 1 lesion while 45 lesions (40%) were identified in the right-sided colon, 27 (24%) in the transverse colon, 28 (25%) in the left-sided colon, and 10 (9%) in the rectum (3 lesions unknown). Endoscopic remission was observed in 24 cases (48%) during the 2 endoscopies prior to lesion detection vs. in 83 controls (42%) for their last 2 endoscopies. Of all CRN lesions, 39 (35%) were found in a colonic segment with confirmed previous endoscopic and/or histologic inflammation during the 1-2 endoscopies prior to lesion detection (31 low-grade and 8 aCRN). Of the 32 patients who underwent (sub)total colectomy or proctocolectomy, 20 (63%) had an indication due to CRN diagnosis. Mortality was 13% (n=32) during follow-up.

**Conclusions:**

The risk for CRN and associated colectomy is high in PSC-IBD. Active mucosal inflammation preceded CRN in the affected colonic segment in only 35% of the lesions. Future expansion and additional analysis of our cohort will focus on the impact of persistent endoscopic remission and impact of biologic therapy on CRN risk in PSC-IBD.

**Funding Agencies:**

None

